# Supplementary figures and images for: Hierarchical association of COPD to principal genetic components of biological systems
Source: PLoS One. 2023 May 25;18(5):e0286064. doi: 10.1371/journal.pone.0286064 (PMC10212185; doi:10.1371/journal.pone.0286064)

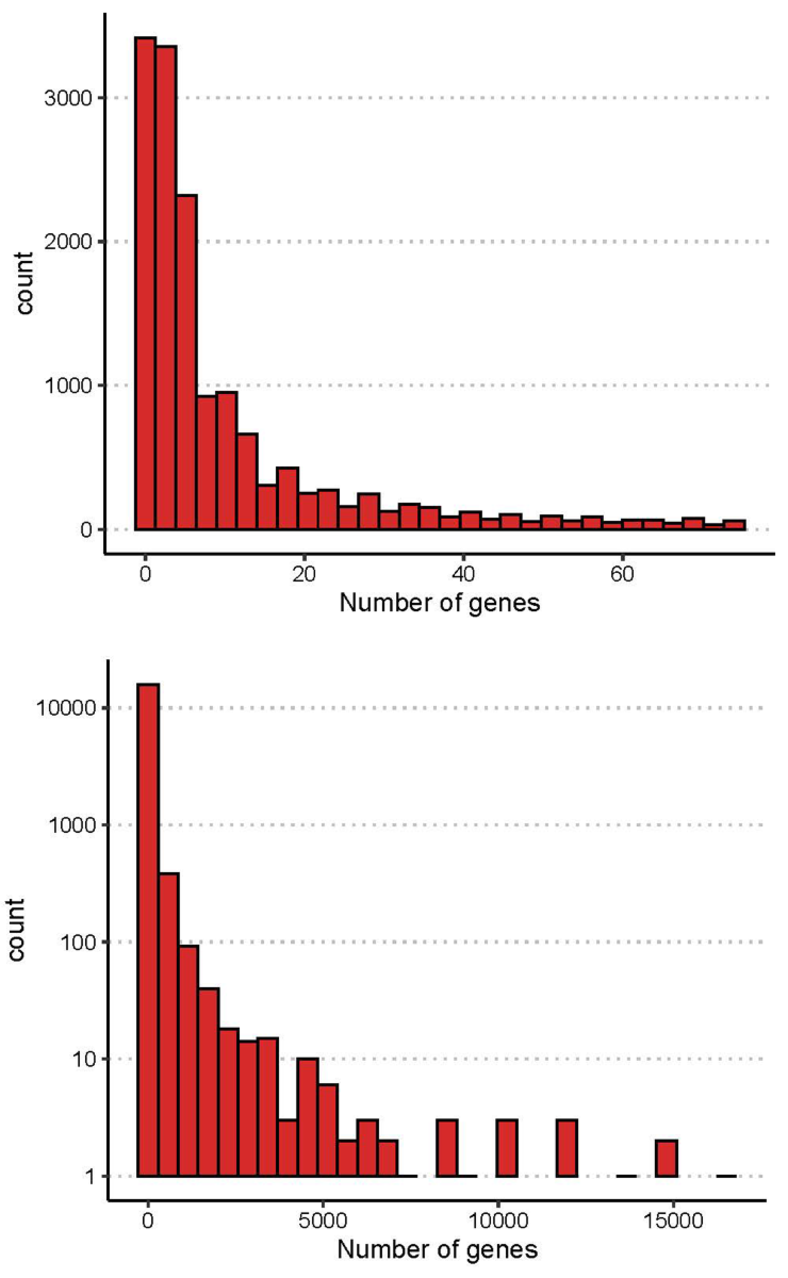

Supplement: S1 Fig — (a) All systems. Horizontal axis is log-scaled. (b) Restricted to systems of up to 75 genes. (TIF) [file pone.0286064.s001.tif]

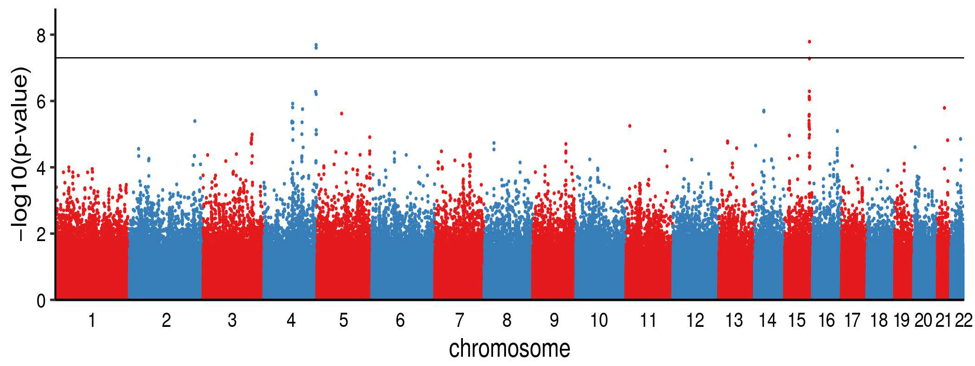

Supplement: S2 Fig — Horizontal line denotes p = 5x10^-8^. (TIF) [file pone.0286064.s002.tif]

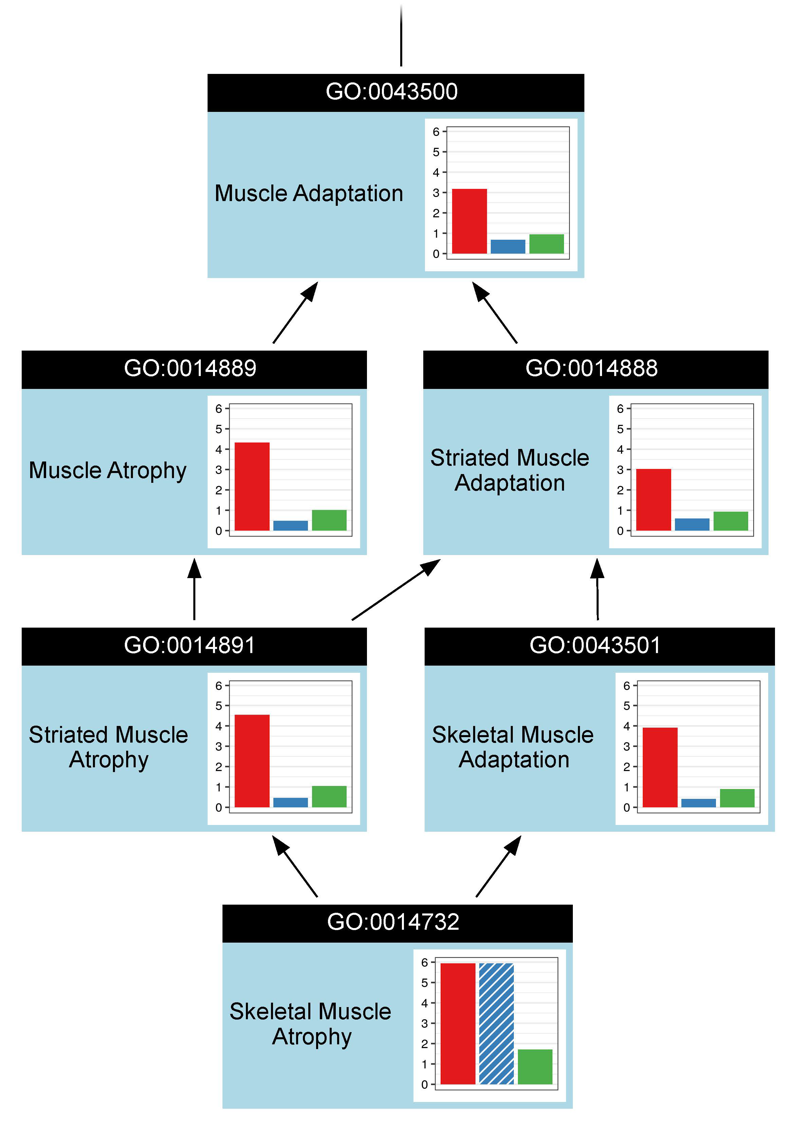

Supplement: S3 Fig — Bar heights indicate -log10(p-value). P-values are unadjusted. Comparison of association results for the original uncorrected analysis (red) and conditional tests (blue- “top child”, green- “top gene”). Shaded bars for the “top child” test indicate the system had no children and thus no correction was applied. (TIF) [file pone.0286064.s003.tif]

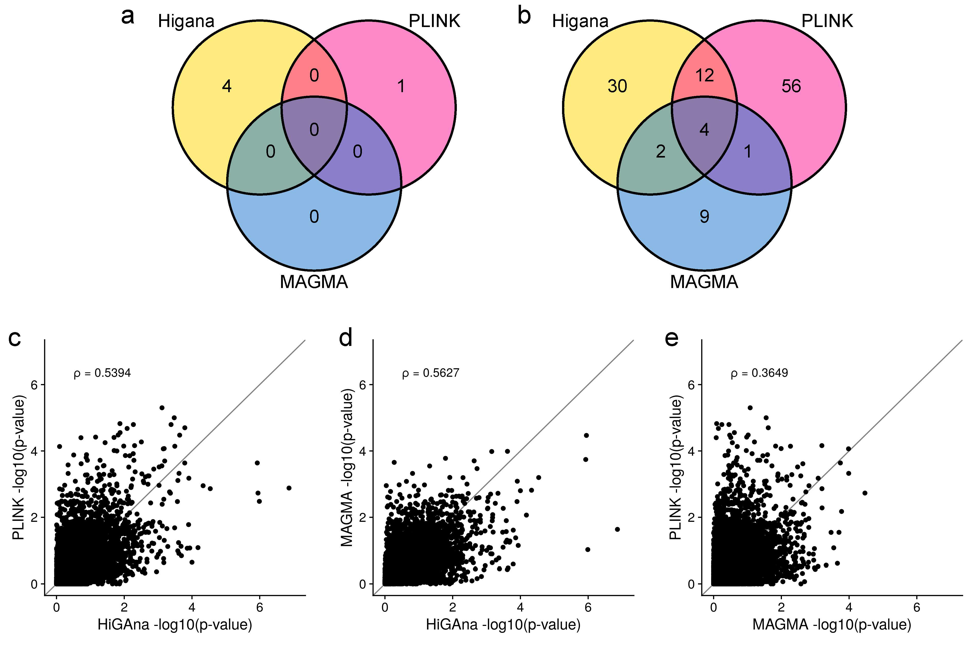

Supplement: S4 Fig — (a) Venn diagram of systems returned at strict FWER < 0.05 for all methods. (b) Venn diagram of systems returned with relaxed p < 0.001. (c-e) Log-scaled p-values for all tested systems. ⍴ is the Pearson correlation coefficient between the log-scaled p-values of each pair of systems. (TIF) [file pone.0286064.s004.tif]

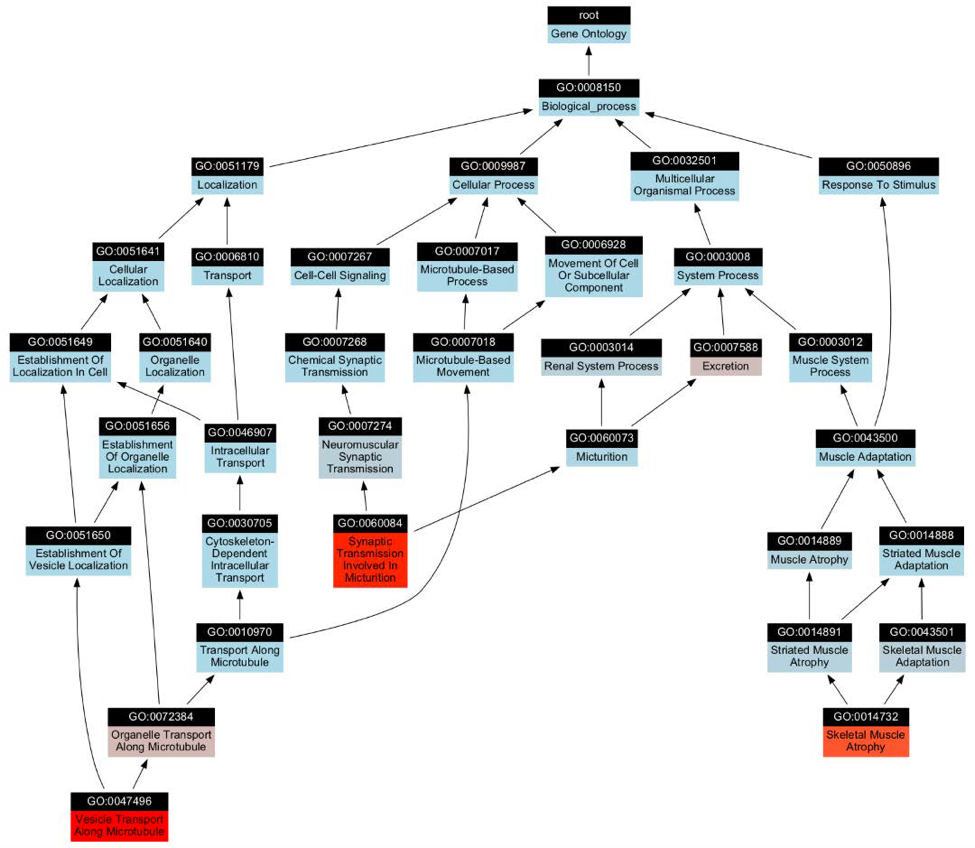

Supplement: S5 Fig — System color corresponds to unadjusted p-value. (TIF) [file pone.0286064.s005.tif]

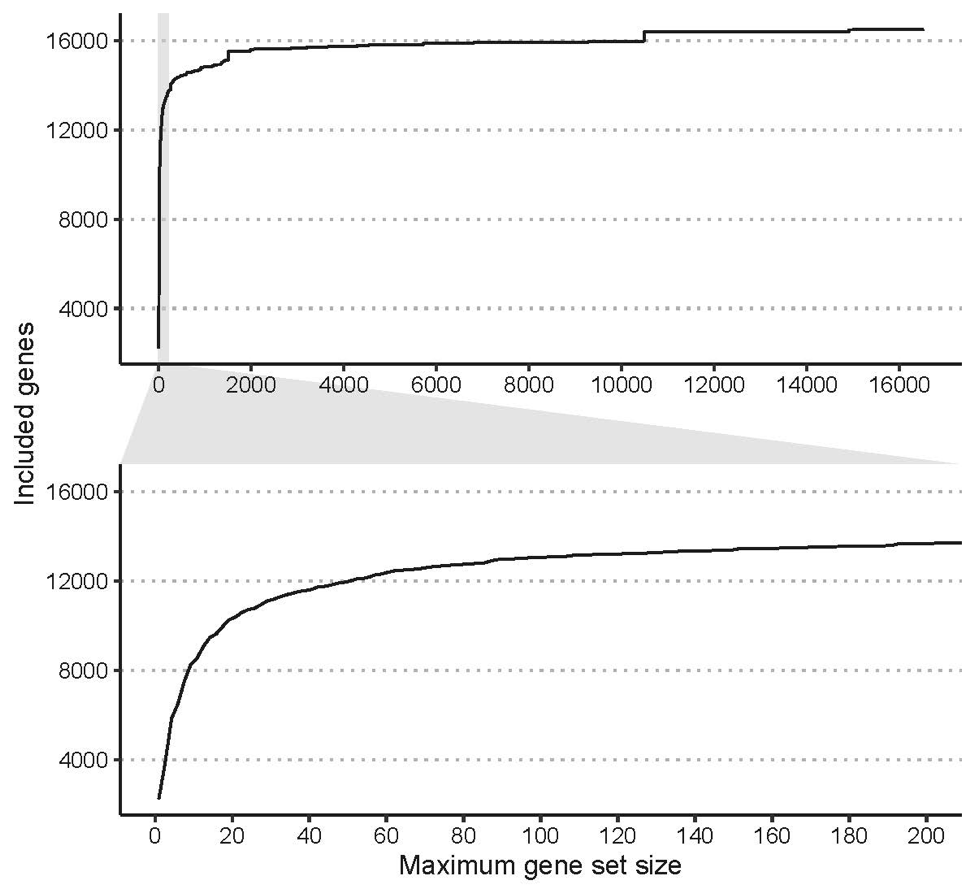

Supplement: S6 Fig — (TIF) [file pone.0286064.s006.tif]

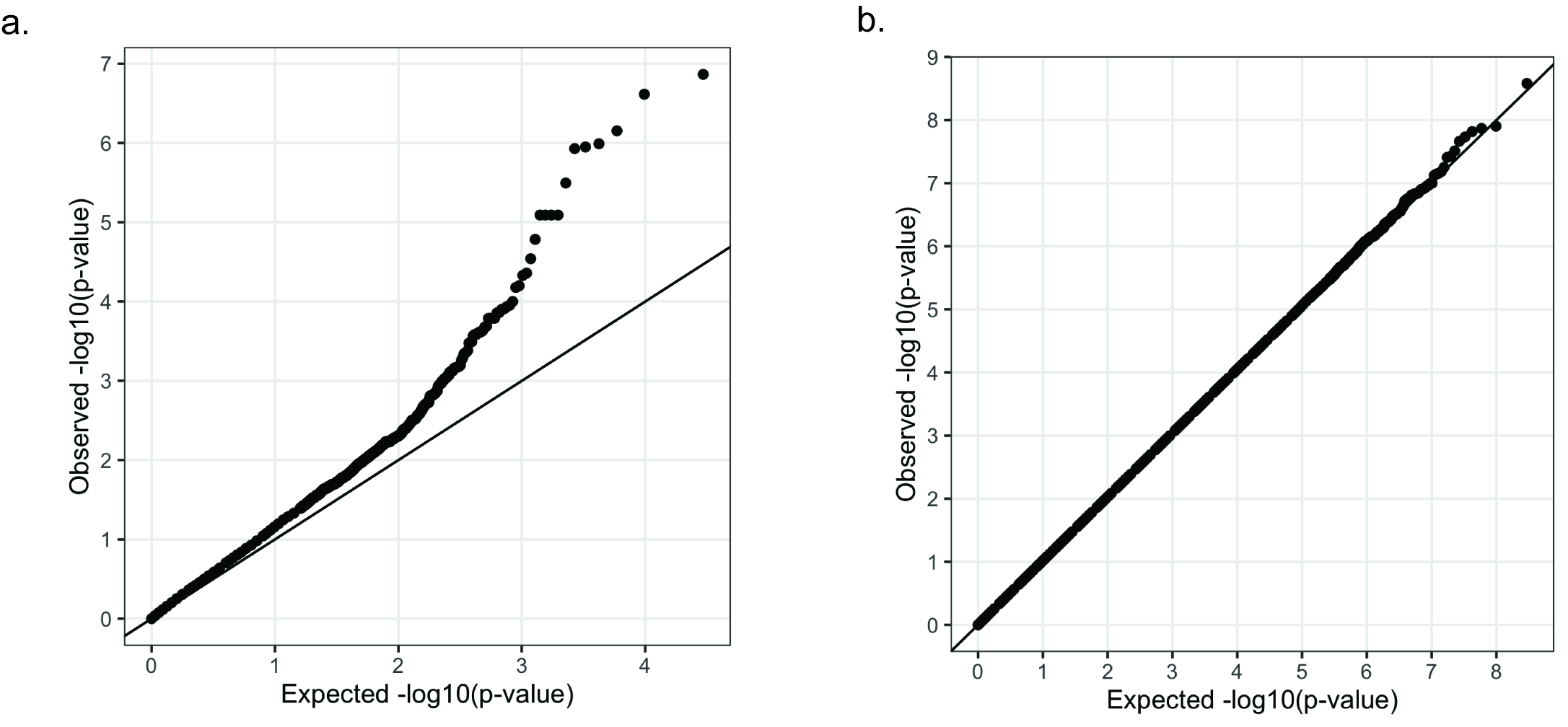

Supplement: S7 Fig — Higana Log QQ-plots (a) The p-values of association of all terms in the COPD non-Hispanic white cohort, plotted against the expected uniform distribution. (b) The p-values of association of all terms from 10000 covariate balanced permutations, plotted against the expected uniform distribution. (TIF) [file pone.0286064.s007.tif]

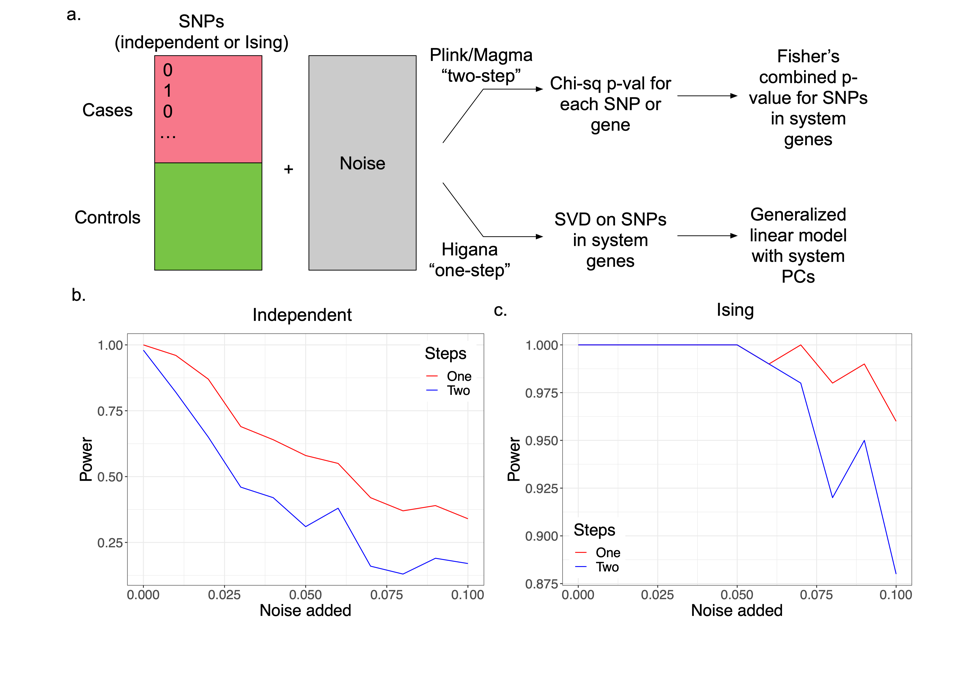

Supplement: S8 Fig — a. Case or control SNPs were simulated using both an independent model or a correlated Ising model. Then these SNPs were passed to either one- and two-step evaluation procedures. b. Power calculation for simulations in the independent and c. Ising models (TIF) [file pone.0286064.s008.tif]
