# Supplementary material for: Hierarchical association of COPD to principal genetic components of biological systems
Source: PLoS One. 2023 May 25;18(5):e0286064. doi: 10.1371/journal.pone.0286064 (PMC10212185; doi:10.1371/journal.pone.0286064)
Supplement: S1 Text — (DOCX) [file pone.0286064.s013.docx]

**Supplemental Text**

**Implementation of other gene set association methods**

As benchmarks for comparison, gene-set association analysis was carried out using two existing tools: the PLINK whole genome analysis toolset (v1.90b3.31) and the tool known as Multi-marker Analysis of GenoMic Annotation (MAGMA, v1.07b). PLINK was invoked using the logistic regression option (--logistic). Genotypes were modeled as an allelic dosage additive effect. Sex, age, smoking status, total pack years, and the first 10 principal components of genotypes were included as additional covariates. In total, the command line invocation of plink was:

plink1.9 --bfile genotypes --logistic perm set-test sex --aperm 5 1000000--covar genotypes.covar --covar-name AGE,SMOKING,PACKYEARS,PC1,PC2,PC3,PC4,PC5,PC6,PC7,PC8,PC9,PC10 --out qc_smoking --set plink_genesets75.txt.

MAGMA first calculates a principal component matrix for each gene, summarizing the LD structure of all SNPs within 10kb, then scores each gene’s association with the phenotype of interest using a standard linear regression model. To score the association of a gene set, each gene p-value is converted to a z-score, and the gene set structure is regressed onto the per gene z-score, yielding a coefficient that indicates each gene sets’ contribution to its constitutive genes’s z-score. The invocation of MAGMA was performed with the following two commands: First the gene level analysis:

magma --bfile qc --gene-model linreg --gene-annot qc.genes.annot --covar file=qc.covar include=AGE,SMOKING,PACKYEARS,PC1,PC2,PC3,PC4,PC5,PC6,PC7,PC8,PC9,PC10 use-sex --out qc_smoking

and then the gene set analysis:

magma --gene-results qc_smoking.genes.raw --set-annot magma_genesets75.txt --model self-contained correct=all --out qc_75_smoking.

MAGMA and PLINK were run using the same gene sets converted to their respective formats that make up the functional hierarchy used by Higana. We measured the computation time needed by each method on a Linux server with 48 cores at 2.50 GHz and 512 GB RAM. PLINK took 5 days 15 hours 23 minutes, Higana took 2 hours 32 minutes, and MAGMA took 2 minutes 29 seconds.
