# Supplementary material for: Hierarchical association of COPD to principal genetic components of biological systems
Source: PLoS One. 2023 May 25;18(5):e0286064. doi: 10.1371/journal.pone.0286064 (PMC10212185; doi:10.1371/journal.pone.0286064)
Supplement: Attachment — Submitted filename: Reviewer response.docx [file pone.0286064.s014.docx]

Dear Editors and Reviewers,

Thank you for your suggestions in how to improve our manuscript. Responses appear in italics.

1. Please ensure that your manuscript meets PLOS ONE's style requirements, including those for file naming. The PLOS ONE style templates can be found at 
<https://journals.plos.org/plosone/s/file?id=wjVg/PLOSOne_formatting_sample_main_body.pdf> and 
<https://journals.plos.org/plosone/s/file?id=ba62/PLOSOne_formatting_sample_title_authors_affiliations.pdf>

*We have modified the document to follow these guidelines*

2. Thank you for including the following ethics statement on the submission details page:
'The COPDGene study protocol was approved by the institutional review boards at the
21 participating clinical centers. All COPDGene participants provided written informed
consent.'

Please also include this information in the ethics statement in the Methods section of your manuscript.

*We have added this text to the Methods section*

3. Thank you for stating the following in the Competing Interests section:

TI is co-founder of Data4Cure, Inc., is on the Scientific Advisory Board, and has an equity interest. TI is on the Scientific Advisory Board of Ideaya BioSciences, Inc., has an equity interest, and receives sponsored research funding. The terms of these arrangements have been reviewed and approved by the University of California San Diego in accordance with its conflict of interest policies. MC is a consultant for Illumina and AstraZeneca. In the past three years, EKS has received institutional grant support from GSK and Bayer.

Please confirm that this does not alter your adherence to all PLOS ONE policies on sharing data and materials, by including the following statement: "This does not alter our adherence to  PLOS ONE policies on sharing data and materials.” (as detailed online in our guide for authors <http://journals.plos.org/plosone/s/competing-interests>).  If there are restrictions on sharing of data and/or materials, please state these. Please note that we cannot proceed with consideration of your article until this information has been declared.

Please include your updated Competing Interests statement in your cover letter; we will change the online submission form on your behalf.

*We have added this to the competing interests and the cover letter.*

4. In your Data Availability statement, you have not specified where the minimal data set underlying the results described in your manuscript can be found. PLOS defines a study's minimal data set as the underlying data used to reach the conclusions drawn in the manuscript and any additional data required to replicate the reported study findings in their entirety. All PLOS journals require that the minimal data set be made fully available. For more information about our data policy, please see <http://journals.plos.org/plosone/s/data-availability>.

Upon re-submitting your revised manuscript, please upload your study’s minimal underlying data set as either Supporting Information files or to a stable, public repository and include the relevant URLs, DOIs, or accession numbers within your revised cover letter. For a list of acceptable repositories, please see <http://journals.plos.org/plosone/s/data-availability#loc-recommended-repositories>. Any potentially identifying patient information must be fully anonymized.

*The minimal data which was used to derive all data in the figures, in the form of system wide association values, are available in data S1-S4.*

Important: If there are ethical or legal restrictions to sharing your data publicly, please explain these restrictions in detail. Please see our guidelines for more information on what we consider unacceptable restrictions to publicly sharing data: <http://journals.plos.org/plosone/s/data-availability#loc-unacceptable-data-access-restrictions>. Note that it is not acceptable for the authors to be the sole named individuals responsible for ensuring data access.

We will update your Data Availability statement to reflect the information you provide in your cover letter.

*We have added the following statement to the cover letter:*

*Due to the patient identifiable nature of the underlying COPDGene data, we cannot directly publish the underlying GWAS data directly. However, the data is available through dbGaP at* [*https://www.ncbi.nlm.nih.gov/projects/gap/cgi-bin/study.cgi?study_id=phs000179.v6.p2*](https://www.ncbi.nlm.nih.gov/projects/gap/cgi-bin/study.cgi?study_id=phs000179.v6.p2)

*We have also added to the Data and materials and Availability:*

***Data and materials availability:*** *A Higana Python implementation is freely available at http://github.com/SimonLarsen/higana All processed data are available in the main text or the supplementary materials. The raw data from the COPDGene study is available from dbGap:* [*https://www.ncbi.nlm.nih.gov/projects/gap/cgi-bin/study.cgi?study_id=phs000179.v6.p2*](https://www.ncbi.nlm.nih.gov/projects/gap/cgi-bin/study.cgi?study_id=phs000179.v6.p2)*, and the subset specifically used for this study is dbGaP Study Accession: phs000296.v5.p2.*

5. Please note that in order to use the direct billing option the corresponding author must be affiliated with the chosen institute. Please either amend your manuscript to change the affiliation or corresponding author, or email us at [plosone@plos.org](mailto:plosone@plos.org) with a request to remove this option.

*Daniel Carlin and Trey Ideker are both associated with UCSD*

6. Please remove your figures from within your manuscript file, leaving only the individual TIFF/EPS image files, uploaded separately.  These will be automatically included in the reviewers’ PDF.

*We have removed all figures from the main text*

Reviewer #1: This study is an very interesting study using a new and experimental bioinformatic method to investigate the relationship between GWAS and COPD. In addition, they found novel results that has not been found in other studies to date.

Reviewer #2: How to incorporate pathway knowledge in genetic association studies has not been yet clear, and authors introduce a concise one-step approach, Hierarchical Genetic Analysis (Higana), which directly computes phenotype associations against each function in the large hierarchy of biological functions documented by the Gene Ontology. With the proposed method, authors identified risk genes and functions for COPD, highlighting microtubule transport, muscle adaptation, and nicotine receptor signaling pathways.

Major comments

1. P-value for the proposed method was based on the permutation. However if there are some correlations between covariates, the permutation-based p-value can be invalidated. For genetic studies, genotypes tend to be independent with other covariates such as age, and sex. However this is very important issue, and authors should provide the solution for this issue.

*The p-values for our methods are not solely based on permutation; in fact the primary association test is a chi-squared test that is not based in permutation at all. This test explicitly handles covariates, using a Chi-squared test to disprove the null hypothesis that the SNPs associated with a system are no more informative than the covariates.*

*Permutation tests were only used for confirmation tests. In this case, we used permutations of the structure of the output labels only, which preserves (and therefore controls or) any correlations of covariates.*

2. Related with the first comment, authors should provide results from the simulated data.

*We have performed simulations with two simulation setups, with knowledge of true positive rates and increasing amounts of noise.*

3. Based on Table 2, authors insisted that Higana and PLINK are much powerful than MAGMA. However it is based on the real data and results in Table 2 cannot be generalized to the general scenario. Empirical power should be compared with the simulated data.

*We have performed simulations and the empirical power curves now appear in the manuscript, as S7 Fig and supporting paragraphs.*

4. It is unclear how to handle the multiple testing problem.

*In this case, multiple testing is not at the level of SNPs, but rather at the level of systems. As such, Bonferroni correction is applied for the number of systems, which is vastly less than the number of SNPs.*

5. I cannot find any link for R package or implementation about Higana. It is better to provide some code.

*As mentioned in the manuscript, the code is available as an R package at* <http://github.com/SimonLarsen/higana/>

6. Permutation-based test is often very computationally intensive for GWAS. For instance, the significance level alpha= 5*10^-8 is often considered and too many replicates are necessary.

*We agree with the reviewer that permutation of patient labels is not viable for the primary test of SNP association significance, for which the (alpha= 5*10^-8) figure pertains, and we avoid all tests of this sort in our method. In fact we use the parameterized chi-squared test as the primary test. However, we do perform two types of permutation tests to evaluate system, rather than SNP-wise, significance: the first is a permutation of the genes associated with each system, which establishes the significance of the system regardless of its position in the hierarchy. As this is a test of the system’s significance, the correct multiple test correction is the number of systems (9312). We also perform a permutation test of the patient labels, but only as confirmation of the system’s significance. Since only four test are necessary (one for each of the identified systems), multiple hypothesis correction is negligible.*

Thank you to the reviewers and editors for their contribution.

~Daniel Carlin, PhD.
