# Supplementary material for: Hierarchical association of COPD to principal genetic components of biological systems
Source: PLoS One. 2023 May 25;18(5):e0286064. doi: 10.1371/journal.pone.0286064 (PMC10212185; doi:10.1371/journal.pone.0286064)
Supplement: Attachment — Submitted filename: Reviewer Response 02062023.docx [file pone.0286064.s015.docx]

Reviewer #1: The responses has been addressed to reviewers comments well. I don't have any more questions and comments.

Reviewer #2: In this article, authors introduce Hierarchical Genetic Analysis (Higana), which directly computes phenotype associations against each function in the large hierarchy of biological functions documented by the Gene Ontology. Using this approach, they identify risk genes and functions for Chronic Obstructive Pulmonary Disease (COPD), highlighting microtubule transport, muscle adaptation, and nicotine receptor signaling pathways. The manuscript is written well. Here are my comments.

1. The p-value was obtained by permutation. However if the covariate adjustment is necessary, then standard permutation cannot be conducted. This problem becomes serious if covariates and SNPs are correlated. This must be taken into account for the permutation.

*There are two types of permutation tests applied here as noted in* ***Empirical assessment of significance via permutation testing****. The ontology permutation test does not disrupt the covariate-SNP relationships and therefore does not need this correction. However, we have now performed the standard label permutation balancing for clinical covarites as noted in the methods, which was used to evaluate the type 1 error rate of the test. The results here are similar to the previous result with the mean number of systems achieving a p-value of 0.05 under the balanced permutation being 0.052suggesting a good control of the type 1 error rate*

2. The authors did not provide QQ plot for Higana analyses, and it is not easy whether Higana controls the type-1 error correctly. It must be provided.

*We Have now included an additional supplemental figure 7 which provide the log qq plot for Higana and the null under balanced permutations as described above. This demonstrates both the inflation of p-values under the experimental condition, and the lack of deviation from the uniform distribution under the permuted null.*

3. Gene-based test can be significant even when only one SNP is significant. Authors showed that 4 pathways are significantly associated with COPD but the p-value for each SNP must be checked.

*We note that in fact we have checked the individual SNPs and genes for significance independently of the systems, and none of them are independently significant, as noted in the results paragraph 2. As an example, the Manhattan plot in Figure 3 shows all loci associated with GO:0047496 Vesicle Transport Along Microtubule highlighted in red, and none of them reach global significance.*

4. Method is not clearly described, and authors should expalin their method clearly.

*We have added some summary explaination in the results and explanation throughout methods to attempt to make our procedure more clear.*

5. If I understand it correctly, all SNPs belonging genes for each pathway are utilized for PCA and PC scores are used for hierarchical association analyses.
If many SNPs are in linkage disequilibrium, and LD block sizes differ by genes, I think that such heterogeneity affcects the statiscal power of the proposed mothod. This problem must be addressed.

*As we note in the discussion, the advantage of collapsing all SNPs genetic signal into PCs is that the variable number of causal SNPs, LD region sizes, and number of genes can all be reduced to a few number of summary variables, thereby providing more homogeneity to the statistical tests. We have added a statement in the discussion to emphasize this point.*

6. It is unclear why the proposed method is statistically more powerful compared to PLINK, and MAGMA. Any discussion is recommended why it performs better

*In paragraph 5 and 6 of the discussion we have sketch out why our method has an advantage over the other methods. These include the detection of multi-locus effects due to combination of SNP effects at a higher organizational level, the increased expressive power of combining effects the logistic regression, and the de-noising effect of PCA analysis.*
